# Safety and feasibility of umbilical cord mesenchymal stem cells in treatment-refractory systemic lupus erythematosus nephritis: time for a double-blind placebo-controlled trial to determine efficacy

**DOI:** 10.1186/ar4677

**Published:** 2014-07-30

**Authors:** Thasia G Woodworth, Daniel E Furst

**Affiliations:** Division of Rheumatology, David Geffen School of Medicine, University of California, Los Angeles, CA USA

## Abstract

As translational clinical researchers familiar with the risk-benefit of hematopoietic stem cell transplantation in autoimmune diseases, we are intrigued by the recent report of umbilical cord mesenchymal stem cell (UC-MSC) transplantation in treatment-refractory systemic lupus erythematosus nephritis by Wang and colleagues. They report the results of an open-label single-arm multicenter phase I/II study. This stimulated us to examine whether collective data from this group provide sufficient evidence for the feasibility, safety, dose rationale, and potential efficacy of UC-MSCs to conduct a randomized controlled trial in such patients. Results, though confounded by variable baseline prednisone and immuno-suppressive treatment, appear to indicate near-term response rates of approximately 50%, which are comparable to those seen with hematopoietic stem cell transplantation but with less morbidity and mortality.

Wang and colleagues have been in the forefront of evaluating the potential for mesenchymal stem cells (MSCs) to treat systemic lupus erythematosus (SLE), based on studies in murine autoimmune disease models, demonstrating immunomodulatory properties of MSCs [1]. We have reviewed the additional reports published in peer-reviewed journals from this group [2–5], together with two protocols available on ClinicalTrials.gov (NCT00698191 and NCT01741857) (Table 1).

**Table 1:** **Overview of open-label phase I/II studies to evaluate mesenchymal stem cells (1 × 10** ^**6**^ **per kg) in treatment-refractory systemic lupus erythematosus**

Authors (date)	ClinicalTrials.gov protocol number	Study design/duration of follow-up	Number of patients	MSC type/regimen	Conditioning	Safety: deaths/serious infection	PD marker ^a^	Efficacy
Sun *et al*. [2]	NR	Single-arm/ median of 8.25 months (range of 3 to 28 months)	16 (15 SLEN)	UC, single infusion	CYC 0.8 to 1.8 mg/kg intravenously, 2 to 4 days	0/0	Percentage of Treg cells increased at 3 months (*P* = 0.03)	‘Decreasing SLEDAI and proteinuria^b^ in all patients’
Liang *et al*. [3]	NCT 00698191	Single-arm/17.2 ± 9.5 months	15 SLEN	BM, single infusion	Included in protocol, but NR	0/0	Percentage of Treg cells increased at 1 week and 3 and 6 months (*P* <0.05)	‘Decreasing SLEDAI and proteinuria^b^ in all patients’
Wang *et al*. [4]	NCT 00698191	Unblinded-randomized, 2-arm/12 months	58 (~88% SLEN)	BM, UC, single versus 2× (7 days apart)	CYC 10 mg/kg per day, day 4, 3, and 2	1/NR	ND	CR single: 16/30 (53%); double: 8/27 (29%)
Wang *et al*. [5]	NR	Single-arm/mean of 27 months	87 (84% SLEN)	BM, UC, single infusion, 18 patients retreated at relapse	CYC 10 mg/ kg/day, day 4, 3, and 2	5/NR	ND	CR in 23/83, relapse 10/83
Wang *et al*. [1]	^c^NCT 01741857	Single-arm	40 (38 SLEN)	UC, 2× infusion, 7 days apart)	No	3/4	ND	MCR 13/PCR 11, 7 relapse

^a^Pharmacodynamic (PD) markers = increased peripheral blood regulatory T (Treg) cells, balanced T helper 1 (Th1)/Th2 cytokines; ^b^ [2] baseline (BL) proteinuria 3.1 (±1.2) g/day versus 3 months, 1.3 (±0.9) g/day (*P* <0.001, n = 15); [3] BL proteinuria 2.7 (±1.2) g/day versus 6 months, 0.9 (±0.8) g/day (*P* <0.01, n = 12); ^c^protocol described at ClinicalTrials.gov consistent with this study, but not explicitly noted in report. BM, bone marrow; CR, complete remission; CYC, cyclophosphamide; MCR, major clinical response; MSC, mesenchymal stem cell; ND, not done; NR, not reported; PCR, partial clinical response; SLEDAI, Systemic Lupus Erythematosus Disease Activity Index; SLEN, systemic lupus erythematosus nephritis; UC, umbilical cord.

Protocol NCT01741857, first posted on 26 November 2012 and updated 1 November 2013, appears to be the protocol for the study recently published in *Arthritis Research & Therapy* [1]. The report largely reflects the protocol, although dose is not described and patient entry criteria required a dose of prednisone of more than 20 mg/day. In the report, only 10 of 40 patients were receiving prednisone of more than 20 mg/day, and one dose - 1 × 10^6^ cells per kg, infused twice 7 days apart - was evaluated. The umbilical cord mesenchymal stem cells (UC-MSCs) for infusion were centrally prepared by using a well-standardized, quality-controlled method, and infusions were well tolerated. One-year mortality (two patients with uncontrolled SLE) and morbidity (five serious infections) compare favorably to those seen with hematopoietic stem cell transplantation in patients with SLE [6].

Previously reported studies by this group [2–5] evaluated stem cells derived from either bone marrow of healthy relatives or umbilical cords donated by healthy consenting mothers in patients with treatment-refractory SLE. Most patients had active SLE nephritis despite receiving prednisone of more than 20 mg and immuno-suppression with cyclophosphamide, mycophenylate mofetil, or leflunomide or a combination of these. In addition, they usually received ‘conditioning’ by cyclophosphamide 0.8 to 1.8 mg/kg per day for 3 days prior to transplant. In contrast, in the recently reported study [1], only UC-MSCs were transplanted, without ‘conditioning’, in patients with active treatment-refractory SLE nephritis receiving a more variable background of prednisone, often less than 20 mg/day, and immunosuppression. Although efficacy is difficult to evaluate because major clinical response (MCR) required that prednisone be tapered to less than 10 mg/day, while maintaining improvements in disease activity measured by British Isles Lupus Assessment Group and Systemic Lupus Erythematosus Disease Activity Index, conditioning is apparently not needed. However, because 18 of 40 patients were receiving prednisone of less than 20 mg/day at the start of the study, the efficacy criterion of ‘tapered’ prednisone is difficult to assess. Although 24 responses - 13 MCR and 11 partial clinical response (CR) - are reported, 6 MCR and 6 partial CR cannot be verified in the reported data, because prednisone tapering appears not to meet criteria for response. In addition, relapse occurred within a year in 6 patients, most receiving at least 20 mg prednisone at baseline. Nevertheless, there may be some evidence for potential efficacy or perhaps corticosteroid sparing, but dose regimen results are not sufficiently clear, or defined by relevant pharmacodynamic activity, to identify a specific UC-MSC dose to use for a randomized controlled trial (RCT).

Review of previous reports may provide some additional insight (Table 1). One open-label study indicates that UC-MSCs and bone marrow mesenchymal stromal cells, each dosed at 1 × 10^6^ cells per kg, are equally effective, based on CR rate [4]. In the same study, patients were randomly assigned in an unblinded manner to receive a single infusion versus two infusions of MSC, 7 days apart; again, no significant difference in CR rate was observed. Of interest are two studies that reported (Table 1) a significant decrease in proteinuria and an increase in peripheral blood regulatory T (Foxp3) cells 3 to 6 months after infusion, potentially providing evidence of a pharmacodynamic effect of MSCs [2, 3]. No correlations with improvement in clinical status were reported, however.

Overall, Wang and colleagues have demonstrated the feasibility of a multicenter trial, as they apparently recruited a sufficient number of patients in a reasonable period of time, and safety was acceptable. Apparently, conditioning pre-MSC dosing is not required, although this aspect of the treatment has not been studied in a controlled manner. It should be pointed out that the collective published results may reflect the fact that some of the same patients were reported in more than one study. For example, two publications [1, 4] refer to the same NCT00698191 protocol; thus, recruitment feasibility may be optimistic. The dose of stem cells, with biologic activity shown in two studies, may have been defined, although it is not absolutely clear (see above caveats). There are also some indications of an appropriate population, but again there seems to be some lack of clarity based on the recently published study [1] where the endpoints were not actually met.

The lack of a well-rationalized dosing regimen, together with a lack of results from an appropriately designed, well-controlled study, makes it extremely difficult to develop a treatment approach with UC-MSCs. Nevertheless, we feel that these results should not be discarded without a proper RCT. Although there may be some challenges in designing a well-controlled, double-blind, placebo-controlled RCT in patients with prednisone-dependent active SLE nephritis, this should be attempted [7].

## Abbreviations

CR: Clinical response
MCR: Major clinical response
MSC: Mesenchymal stem cell
RCT: Randomized controlled trial
SLE: Systemic lupus erythematosus
UC-MSC: Umbilical cord mesenchymal stem cell.
